# Does CaMKII decode Ca^2+^ oscillations?

**DOI:** 10.1186/1471-2202-13-S1-O15

**Published:** 2012-07-16

**Authors:** Thiago M Pinto, Maria J Schilstra, Volker Steuber

**Affiliations:** 1 Science and Technology Research Institute, University of Hertfordshire, Hatfield, Herts, AL10 9AB, UK

## 

Ca^2+^/calmodulin-dependent protein kinase II (CaMKII), which is present in high concentrations in the brain, contributes to many forms of synaptic plasticity. The induction of synaptic plasticity by CaMKII involves an intracellular signalling cascade that links neuronal Ca^2+^ signals with the phosphorylation of neurotransmitter receptors; an important step in this biochemical cascade is the autophosphorylation of CaMKII after binding of Ca^2+^/calmodulin (Ca_4_-CaM).

The dependence of this autophosphorylation reaction on the temporal structure of Ca_4_-CaM signals has been investigated in previous experiments [1] and computer simulations [2]. These experimental and theoretical studies have indicated that the autophosphorylation of CaMKII is sensitive to the frequency of repetitive Ca^2+^ pulses, and it has been concluded that CaMKII can decode oscillatory Ca^2+^ signals [1,2].

Here, we apply a simplified version of the commonly used CaMKII activation model by Dupont and collaborators [2] to investigate the mechanism that underlies the dependence of the overall autophosphorylation kinetics on the frequency of Ca^2+^ oscillations. In the simulations by Dupont et al., CaMKII was subjected to different average, or 'effective', Ca_4_-CaM concentrations, which in turn affected the average concentration of the CaMKII subunits, and the autophosphorylation kinetics.

**Figure 1:**
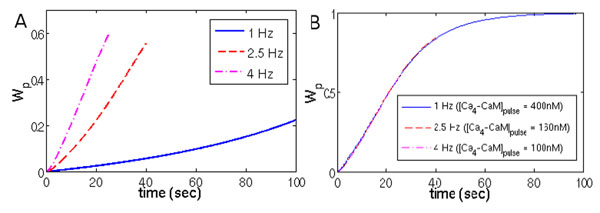
CaMKII phosphorylation and its dependence on the effective Ca_4_-CaM concentration. **(A)** Temporal evolution of the phosphorylated form of CaMKII (Wp) in response to one hundred 200 ms square pulses of Ca_4_-CaM (100 nM) at frequencies of 1 Hz (solid blue), 2.5 Hz (dashed red) and 4 Hz (dashed-dotted magenta) in our simplified model. **(B)** Wp in response to one hundred 200 ms square pulses of Ca_4_-CaM at 1, 2.5 and 4 Hz, but with scaled pulse amplitudes so that the effective concentration of Ca_4_-CaM is 80 nM. The amplitudes of Ca_4_-CaM pulses are 400 nM at 1 Hz (solid blue), 160 nM at 2.5 Hz (dashed red) and 100 nM at 4 Hz (dashed-dotted magenta).

We first replicate the simulation results presented in [2] with our simplified model (Figure 1A). To identify the mechanism that underlies the observed frequency dependence, we then rescale the Ca_4_-CaM concentrations to an equal effective concentration, and compare the phosphorylation kinetics (Figure 1B). We demonstrate that in our model the overall phosphorylation rate under sustained application of Ca_4_-CaM pulses depends on the average (‘effective’) concentration of Ca_4_-CaM in the system, rather than on the pulse frequency itself. Moreover, we show that the application of a constant level of Ca_4_-CaM with the same mean concentration as in the pulsed protocol results in the same level of CaMKII phosphorylation.

Our simulation results indicate that the notion of CaMKII as a decoder of Ca^2+^ oscillations is misleading and suggest experimental tests with rescaled Ca_4_-CaM concentrations.
